# Supplementary material for: Anti-Predation Responses to Conspecific versus Heterospecific Alarm Calls by the Nestlings of Two Sympatric Birds
Source: Animals (Basel). 2022 Aug 22;12(16):2156. doi: 10.3390/ani12162156 (PMC9404724; doi:10.3390/ani12162156)
Supplement: Supplementary file 1 [file animals-12-02156-s001.zip › animals-1846098-supplementary.pdf]

Supplementary data

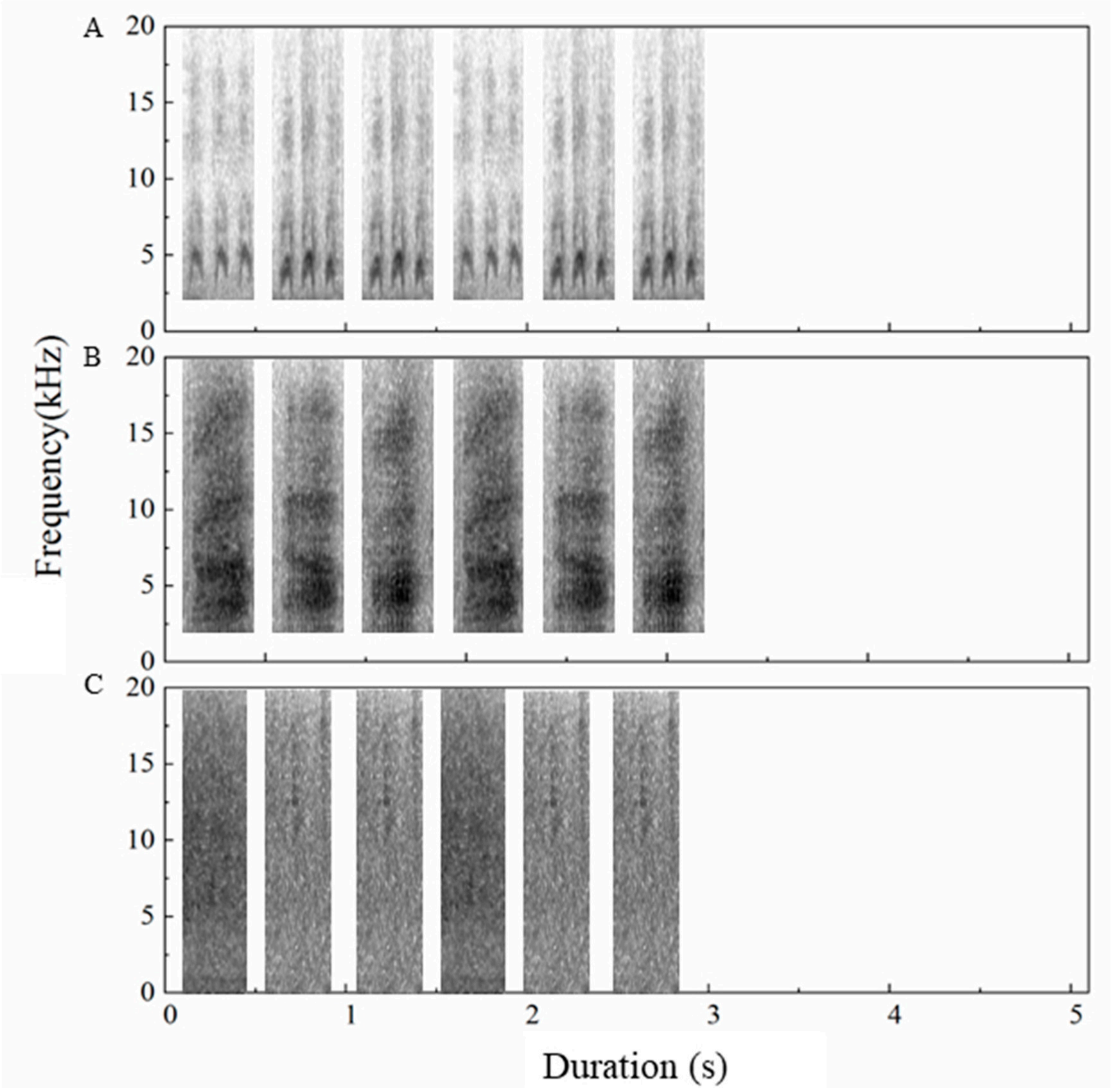

Figure S1. Sound spectrograms of (A) the mobbing alarm calls of vinous-throated parrotbills (VP), (B) the mobbing alarm calls of oriental reed warblers (ORW), and (C) background noise.
